# Protein Kinase Targets in Breast Cancer

**DOI:** 10.3390/ijms18122543

**Published:** 2017-11-27

**Authors:** Marilina García-Aranda, Maximino Redondo

**Affiliations:** 1 Biochemistry Department, Hospital Costa del Sol, REDISSEC, Carretera de Cádiz km, 187, 29600 Marbella, Málaga, Spain; marilina@uma.es; 2 Biochemistry Department, Facultad de Medicina de la Universidad de Málaga, Bulevar Louis Pasteur 32, 29010 Málaga, Spain

**Keywords:** breast cancer, kinases, phosphatases, target

## Abstract

With 1.67 million new cases and 522,000 deaths in the year 2012, breast cancer is the most common type of diagnosed malignancy and the second leading cause of cancer death in women around the world. Despite the success of screening programs and the development of adjuvant therapies, a significant percentage of breast cancer patients will suffer a metastatic disease that, to this day, remains incurable and justifies the research of new therapies to improve their life expectancy. Among the new therapies that have been developed in recent years, the emergence of targeted therapies has been a milestone in the fight against cancer. Over the past decade, many studies have shown a causal role of protein kinase dysregulations or mutations in different human diseases, including cancer. Along these lines, cancer research has demonstrated a key role of many protein kinases during human tumorigenesis and cancer progression, turning these molecules into valid candidates for new targeted therapies. The subsequent discovery and introduction in 2001 of the kinase inhibitor imatinib, as a targeted treatment for chronic myelogenous leukemia, revolutionized cancer genetic pathways research, and lead to the development of multiple small-molecule kinase inhibitors against various malignancies, including breast cancer. In this review, we analyze studies published to date about novel small-molecule kinase inhibitors and evaluate if they would be useful to develop new treatment strategies for breast cancer patients.

## 1. Introduction

### 1.1. Breast Cancer

Breast cancer is the most commonly diagnosed malignancy and the second leading cause of cancer death among women worldwide [1].

Although, nowadays, most breast cancers are diagnosed early enough to be successfully treated with surgery, chemotherapy, radiotherapy, or a combination thereof, a significant percentage of patients will not respond to these treatments and end up with metastatic disease to bone, lung, liver, brain [2], or other body organs, ultimately causing a patient’s death [3], which justifies the search of new therapeutic strategies.

#### Targeted Therapies

Among the new treatments developed in recent years, targeted therapies have been a milestone in fighting cancer, because, contrary to non-specific cytotoxic agents against dividing cells, these therapies are based on the use of drugs, or other substances, especially designed to interfere with molecules related with tumor growth and progression.

Over the past decade, many studies have shown a causal role of protein kinase dysregulations or mutations in different human disorders, including Alzheimer’s and Parkinson’s disease, diabetes, atherosclerosis, stroke, and inflammatory diseases (rheumatoid arthritis, Crohn’s disease) [4], which made them particularly sensitive to appropriate protein kinase inhibitors.

Along these lines, cancer research has proven that multiple protein kinases play an important role during human tumorigenesis and cancer progression, turning these molecules into valid candidates for the development of new targeted therapies, and finally leading to the discovery and introduction in 2001 of imatinib as treatment for chronic myelogenous leukemia. This discovery revolutionized research in genetic pathways that are related to tumor proliferation and improved our knowledge of various protein kinases with a crucial role in different types of cancer, which eventually led to the development of several small-molecule kinase inhibitors against different malignancies, including breast cancer.

### 1.2. Protein Kinases

#### 1.2.1. Protein Kinases Classification

Human protein kinases (PK) constitute a large family of enzymes, known as the human kinome, which are encoded by about 1.7% of all human genes [5]. According to the hydroxy-amino-acid target that these enzymes phosphorylate in their substrates, members of protein kinase superfamily have been classically classified into two main groups: Serine-Threonine kinases, which phosphorylate serine or threonine amino acids, and Tyrosine kinases (TKs), which phosphorylate tyrosine amino acids. A third group, consisting on dual-specificity protein kinases has also been proposed, as they are able to phosphorylate both tyrosine and serine/threonine residues [6].

The first protein kinases to be identified were tyrosine kinases, which have been well described. According to their location in the cell, tyrosine kinases are classified into:

- Transmembrane receptor kinases, with a ligand-binding extracellular domain and a catalytic intracellular kinase domain.
- Non-receptor tyrosine kinases, lacking the transmembrane domains and located in the cytosol, nucleus, or the inner surface of plasma membrane [4].

**Figure 1:**
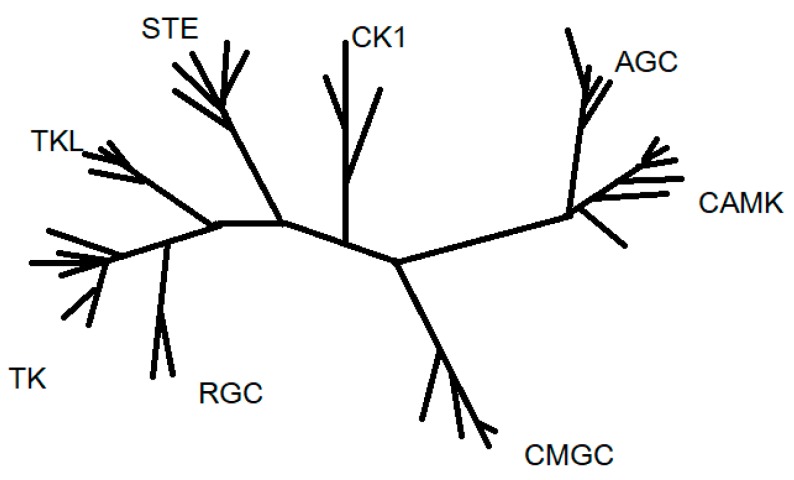
Proposed phylogeny general outline for the kinase superfamily [5]. The phylogenetic tree of human kinome, depicts the relationship between different members of protein kinase-superfamily based on homologies in their catalytic domains.

Protein kinases are also classified regarding catalytic domain sequence comparisons into eight main families: AGC (A, G and C protein kinases), CAMK (Ca^2+^/CAM-dependent protein kinases), CK1 (casein kinase 1), CMGC (CDK, cyclin-dependent kinases; MAPK, mitogen-activated protein kinases; GSK3, glycose synthase kinase-3; CLK, cdc2-like kinases), RGC (receptor guanylate cyclase), STE (homologues of yeast sterile 7, 11, 20 kinases), TKs (tyrosine kinases), and TKL (tyrosine kinases-like protein kinases). This classification also includes many atypical kinases that are lacking sequence similarity to the conserved eukaryotic protein kinase catalytic domain [4] (Figure 1).

#### 1.2.2. Protein Kinases Function

**Figure 2:**
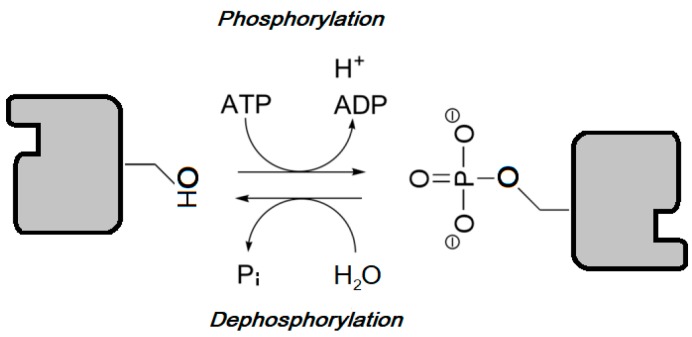
Via phosphorylation, protein kinases chemically transfer γ-phosphate from ATP (or GTP) to a targeted amino acid with a free hydroxyl group from its protein substrate. Protein phosphatases catalyze the opposite reaction, transferring the phosphate from a phosphoprotein to a water molecule.

Along with phosphatases, protein kinases are included in the phosphorus transferases group, enzymes that catalyze the reversible transfer of phosphate between their substrates Figure 2.

The activation of a surface kinase receptor by its ligand, or other stimulus, generally entails the activation of non-receptor kinases cascade reactions, which results in the translation of extracellular and intracellular signals throughout the cytoplasm and the nucleus. Thereby, the activation of genes and the cellular response is controlled through the coordinated, but independent, work of protein kinases, phosphatases, and the phosphorylation status of their substrate proteins.

The phosphorylation processes can induce conformational changes in the substrate protein, resulting in the disruption or creation of protein-protein interaction surfaces. These conformational changes condition protein activity, cellular location, or association with other proteins [7]. The importance of protein post-translational modifications that are catalyzed by these enzymes becomes apparent when considering that although protein kinase genes in most eukaryotes constitute up to only 2% of the genome, protein kinases can phosphorylate more than 30% of cellular proteins [8]. Actually, since the first characterization of protein kinase activity in the 1950s [9], these enzymes have been shown to regulate molecular pathways that are essential for most cellular processes, including proliferation, metabolism, migration, survival, and apoptosis.

An uncontrolled kinase activity, as a result of activating mutations or the loss of inhibitory mediators, for example, is commonly found in human cancer [10], leading to cellular proliferation, differentiation, and apoptosis inhibition [11]. In this respect, different kinases [12] have been considered to be oncogenic, as their transforming activity can determine the survival and proliferation of cancer cells [13].

#### 1.2.3. Small Molecule Kinase Inhibitors

Small molecule inhibitors have become a valuable tool in the development of targeted therapies against cancer, as they are highly selective and effective against specific protein kinases [14], and they present a more favorable side effect profile than conventional cytotoxic chemotherapy [15]. So much so, that in recent years over 130 kinase-specific inhibitors have been in phase 1–3 clinical trials [16], and a number of them have been approved for clinical use.

Protein kinases present a core, which is common to all protein kinases, which is required for catalytic activity. This core contains a conserved structure and sequence incorporating a glycine-rich N-terminal ATP-binding pocket and a central conserved aspartic acid residue [17,18]. For this reason, tyrosine kinase inhibitors are designed as non-peptide anilino-quinazoline compounds, homologous of the adenosine trisphosphate (ATP), with the ability to compete for the ATP-binding domain of the cytoplasmic catalytic kinase domain [19]. As a result, these molecules prevent kinase phosphorylation and the subsequent downstream signal transduction pathways activation, which finally leads to an enhanced apoptosis and a decreased cellular proliferation [11].

Since the design of highly selective small molecules with valid pharmaceutical properties is based on the presence of mutated or over-expressed kinases [20], and given that these enzymes’ catalytic core is highly conserved, one of the major challenges in the design of small kinase inhibitors is to prevent a given substance from exerting its action on another kinase (Table 1).

However, the great challenge in the development of effective kinase inhibitors lies in the complexity of kinome dynamics, since kinase networks are well interrelated and the inhibition of one kinase can be bypassed by the activation of alternate kinases. As a result, cells become resistant to single agents [12] and the use of kinase inhibitors in combined therapies with other drugs is needed [22].

## 2. Protein Kinase Targets for Breast Cancer Treatment

### 2.1. Breast Cancer Clinical Classification and Standard of Care

Thanks to the Human Genome Project and to the deep sequencing of breast cancer genome and transcriptome, this heterogeneous disease, which may be a reflection of distinct etiologic pathways [23], has been clinically classified into at least four major molecular subtypes of invasive breast cancer (HER2-enriched, luminal A, luminal B and basal-like) [23,24], with different prognosis and treatment response [20,23,25].

#### 2.1.1. HER2-Enriched

Human epidermal growth factor receptor 2 (HER2)-enriched mammary tumors have been extensively studied and are well described. Together with HER1 (EGFR/ErbB-1), HER3 (ErbB-3), and HER4 (ErbB-4), HER2 (ErbB2/neu) is a member of the ErbB membrane tyrosine kinase receptors family, tightly related to the transcription of signaling pathways leading to cell proliferation, differentiation and apoptosis inhibition [11,19].

HER2 is constitutively activated in approximately 20–30% of breast cancers [11,12]. As HER2 overexpression is widely known to dysregulate cell proliferation in the aggressive HER2-positive breast cancers, this protein constitutes an important therapeutic target for patients with this cancer subtype [26].

The approved therapy for HER2-driven breast cancer is the humanized monoclonal antibody trastuzumab (Herceptin) that binds to and blocks the extracellular domain of HER2. Although trastuzumab has dramatically improved the outcome for patients with this type of cancer, resistance development is a recurrent problem that has motivated the search of alternative therapies targeting this tyrosine kinase receptor. In this regard, the use of small kinase inhibitors like lapatinib/Tykeb, neratinib [27], gefitinib [11], or afatinib [28] have shown preclinical and clinical evidence in the treatment of HER2-enriched tumors (Table 2).

#### 2.1.2. Hormone Receptor Positive (Luminal-A, Luminal-B)

Both luminal-A and luminal-B breast cancer subtypes, which account for up to 75% of breast tumor cases [36,37,38], are hormone receptor-positive (HR-positive), and, therefore, they express estrogen receptors (ER), progesterone receptors (PR), and/or estrogen-responsive and ER-dependent gene products [39].

In luminal-A and luminal-B breast cancers, the estrogen hormone (17β-estradiol) plays a major role during different hallmarks of cancer [36], including apoptosis regulation, cell proliferation, and the expression of growth factors and their receptors [40]. For these reasons, luminal-A and luminal-B breast carcinomas are, theoretically, sensitive to hormone-targeted treatments. Indeed, endocrine therapy has a proven effectiveness of approximately 50–60% [36,41], being Tamoxifen (TMX, Nolvadex) the most common drug used in clinical practice over the past decades as first-line treatment in pre- and post-menopausal women with ER-positive breast cancer [42]. However, although this competitive ER-receptor antagonist has shown a significant reduction of recurrence (40–50%), and the risk of death from breast cancer (30–35%) [36], the existence of an important number of cases with natural or acquired resistance to tamoxifen along with long-term toxicities [39] has motivated the search for new approaches for HER2-enriched breast cancer patients (Table 3).

These novel compounds have demonstrated superior efficacy, reduced incidence of endometrial cancer, reduced blood clot formation, as well as a prolonged disease-free survival [40] time to recurrence and time to distant recurrence when compared to tamoxifen [44,45], representing the recommended standard therapy for post-menopausal women with ER-positive breast cancer nowadays [43,44].

Despite these positive effects, aromatase inhibitors also induce increased bone loss, bone pain [40], and a significant percentage of patients (range 30% to 65%) present primary or secondary resistance to them [45], which are reasons that have justified the continued search of alternatives to hormone-therapies.

The mechanism by which ER-positive breast cancers become estrogen independent to long-term estrogen deprivation and start to grow is under intense study and remains poorly understood. Findings in this regard include the deregulation of components of the ER pathway itself, alterations in cell cycle and cell survival signaling molecules, and the activation of alternative signaling pathways that promote the resistance to endocrine therapies [38]. Studies carried out in this area show an association between ER-α expression and the activity of several kinases and phosphatases [37,38,46,47,48]. Many protein kinase-encoding genes also appear to be altered in ER-positive breast tumors [41,49], which has opened the possibility of developing treatment strategies for these tumors that are based on the targeted inhibition of altered kinases.

#### 2.1.3. Basal-Like

In contrast with HER2 and ER-positive breast carcinomas, basal-like breast tumors are characterized by a gene-expression profile that is similar to that of the basal-myoepithelial layer of the normal breast along, with the absence of HER2 overexpression and the absence or low levels of estrogen receptor expression [50].

The triple negative breast cancer (TNBC) subtype, which constitutes approximately 80% of the basal-like tumors, accounts for approximately 10–15% of breast carcinomas, and is characterized by the lack of expression of both hormone receptors (estrogen and progesterone) and HER2-receptor over-expression [51]. For these reasons, both TNBC and basal-like breast cancers usually lead to an aggressive disease, with a high probability of metastasis [50], and with poor prognosis, which is due, in part, to the absence of an existing effective targeted therapy.

### 2.2. Altered Protein Kinases in Breast Cancer

The characterization of each breast cancer subtype has usually been associated to the identification of mutated or altered kinases [41]. Indeed, as each breast cancer subtype can present a unique expression profile of protein kinases that can be targeted by small molecule kinase inhibitors [20,49], the way to address each type of breast tumor must be different, therefore becoming necessary to carry out a previous study of each patient in order to choose the most appropriate treatment.

Understanding the role of protein kinases during different hallmarks of breast cancer is not an easy task due to the complexity of the interconnections between different routes. Indeed, tumor cells can respond to anti-tumor treatments and become resistant through feedback and crosstalk between different signaling pathways, usually involving protein kinases that are common to different breast cancer subtypes. Therefore, approximately 20% of all breast carcinomas will overexpress HER2 receptor and 40–50% of these will also be ER-positive [41]. These ER/HER2-positive tumors are more likely to develop resistance to tamoxifen and present ligand-independent activation of ER-signaling [41]. Likewise, secondary resistance to aromatase inhibitors also occurs after a switch from dependence on ER signaling to growth factor-mediated pathways, like HER2 dependence [45].

The most relevant results obtained in the protein kinase target search for breast cancer treatment are presented below.

#### 2.2.1. PI3K/Akt/mTOR Signaling Pathway

Multiple research studies are currently focused on the potential use of the phosphatidylinositol-3-kinase (PI3K)/Akt (protein kinase B)/mammalian (or mechanistic) target of rapamycin (mTOR) signaling pathway as a new target against cancer.

PI3K/Akt/mTOR pathway hyperactivation has been implicated in breast cancer tumorigenesis and resistance to endocrine therapy in ER-positive carcinomas [52] and trastuzumab in HER2-positive carcinomas [53]. Besides, since PI3K/Akt/mTOR pathway activation has also been related to cell proliferation, survival, adhesion, migration, invasion, altered metabolism, deregulated apoptosis, angiogenesis [54,55], anoikis [56], as well as to breast cancer progression and response to therapy [55,57], this pathway has become one of the main targets to restore the sensitivity of resistant breast tumors.

The biological significance of this complex pathway has been thoroughly investigated and extensively reviewed (Table 4).

#### 2.2.2. Phosphatase and Tensin Homologue (PTEN)

The phosphatase and tensin homologue deleted on chromosome 10 (PTEN), which is a phosphoinositide that can inhibit cellular proliferation, survival, and growth by inactivating PI3K-dependent signaling [64], is one of the most frequently disrupted tumor suppressors in human cancer [65] (Table 5).

Interestingly, some studies about the effect of PTEN expression on HER2-enriched breast carcinomas show contradictory results. In this regard, although some studies show that reduced levels of PTEN do not correlate with a high Ki-67 index value, cellular proliferation inhibition, nor Akt phosphorylation control [72], additional research show that PTEN expression correlates to longer overall survival [73]. Studies also show that PTEN inactivation can indirectly promote PI3K de-regulation and help maintain advanced HER2-positive breast cancer disease [60], which is associated with poor clinical outcome [74] and accelerated breast cancer progression [60]. The cause of these contradictory results seems to be due to the lack of standardization of PTEN status determination [75], making it necessary for additional investigations in this respect.

Although PTEN status apparently makes no significant differences in the clinical outcome of TNBC patients [74], these malignancies show a tumor protein p53 deficiency [76,77] and a low PTEN expression [74,76,78], which is also associated with early-onset breast cancer, late stage and high levels of IGFBP2 (insulin like growth factor binding protein 2) [71].

#### 2.2.3. PDK1

Phosphatidylinositol 3-kinase/phosphatidylinositide-dependent protein kinase 1 (PDPK1, PDK1) is a master kinase key for the activation of Akt and many other AGC kinases, with an important role during the activation of cancer cell proliferation and survival pathways [79] (Table 6).

#### 2.2.4. Mitogen-Activated Protein Kinase Pathway

Mitogen-activated protein kinase (MAPK, MAPK/ERK, Ras-Raf-MEK-ERK) pathways comprise a module of three conserved and sequentially activated protein kinases. MAPK has a significant role during the transduction of extracellular signals to different pathways regulating fundamental processes, such as cell growth, proliferation, differentiation, development, transformation, migration, or death [81,82,83]. Under normal conditions, MAPK is tightly regulated by phosphatases and bidirectional communication with other pathways, such as Akt/mTOR pathway [84].

Different MAPKs, such as extracellular signal-regulated kinases (ERK)1/2, ERK3/4, ERK5, ERK7/8, Jun N-terminal kinase (JNK)1/2/3 and the p38 isoforms α/β/γ (ERK6)/δ, have been characterized in mammals [82]. Pathways involving ERK-1 and -2 are amongst the most relevant to malignant breast cancer behavior [85] and cross-talk between ER-α and MAPK signaling pathways has also been pointed to as key oncogenic axis responsible for the development of estrogen-independent growth of breast cancer cells that are initially ER-α positive and hormone sensitive [37].

Though recent studies have evidenced that breast carcinomas frequently contain an increased proportion of cells with the activated form of MAPK [86], the precise relationship between MAPK activation and tumor proliferation, apoptosis, degree of invasiveness, and disease free and overall survival is still under study [86]. Indeed, although de-regulation of MAPK signaling can lead to the development and progression of cancer [82], recent evidence indicates that the MAPK/ERK signaling node can also function as a tumor suppressor [84], which justifies additional research in this regard (Table 7).

#### 2.2.5. Cell Cycle Proteins or Mitotic Kinases

Mitotic kinases, including CDK (cyclin-dependent kinase), Aurora (AURK), Polo-like (PLK) and NIMA (Never In Mitosis)-related kinase families play an important role as regulators of cell division and cytokinesis and their dysregulation has been related to tumorigenesis [90,91] (Table 8).

##### Cyclin-Dependent Kinases (CDK)

Mammalian cell cycle is tightly regulated by cyclins and their associated cyclin-dependent kinases (CDKs) [92], a family of serine/threonine kinases whose dysregulation has been found in a majority of human cancers [92,108,109], accelerating cell division and malignant transformation [110].

##### Aurora Kinases

Aurora kinases constitute a collection of highly conserved serine/threonine kinases that control the accurate and equal segregation of genomic material during mitosis [91]. Three aurora kinase members have been identified in mammals: Aurora kinase A (AURKA), aurora kinase B (AURKB, an important mitotic kinase involved in chromosome segregation and cytokinesis [111]), and aurora kinase C (AURKC), whose over-expression has been related in a greater or lesser degree to many types of malignancies, including breast cancer [91].

Although the role of AURKC during carcinogenesis is still unclear, the effects of AURKA over-expression have been well described. In this respect, it has been suggested that constitutive activation of Raf-oncogenic signaling induces the stabilization and accumulation of AURKA [96]. Nuclear accumulation of AURKA has an oncogenic role [112], as it is related to breast cancer progression through the development of centrosome amplification, chromosome instability [37], and transition from epithelial to a highly invasive mesenchymal phenotype [96]. In this regard, although there are studies showing that there is no difference between AURKA expression in primary metastatic breast carcinomas when compared to control cases [113], AURKA expression levels have been proposed as a useful prognostic marker for patients with ER-positive, normal-like, and luminal A or B-type breast cancer tumors [114].

##### Polo-Like Kinase 1 (PLK1)

Polo-like kinase 1 (PLK1) is a serine/threonine protein kinase that plays an important role in the initiation, maintenance, completion of mitosis [102], and maintenance of genomic stability [103]. PLK1 over-expression has been related to human cancer, which is usually associated with poor prognosis [102].

##### NIMA (Never in Mitosis)-Related Kinases

This polymorphic family of kinases belongs to the “Other” kinase group, and includes NIMA-related kinase 2 (NEK2), which is a serine/threonine kinase that is involved in the regulation of centrosome duplication and spindle assembly during mitosis [106].

#### 2.2.6. Sphingosine Kinases

Sphingosine kinase (SK, SphK) isozymes are a class of G protein-coupled receptor kinases (GRK) that catalyze the phosphorylation of sphingosine into sphingosine-1-phosphate (S1P). Different sphingolipid metabolites are second lipid messengers that are involved in diverse cellular processes, including migration, proliferation, and apoptosis, having been identified as biomarkers in different types of cancer by promoting angiogenesis and tumorigenesis [115].

Over-expression of sphingosine kinase 1 (SK1), which has been causally associated with breast cancer progression and resistance to drug therapies [116,117,118], is predictive of poor prognosis in human breast cancer [119] (Table 9).

### 2.3. Targeted Studies

**Figure 3:**
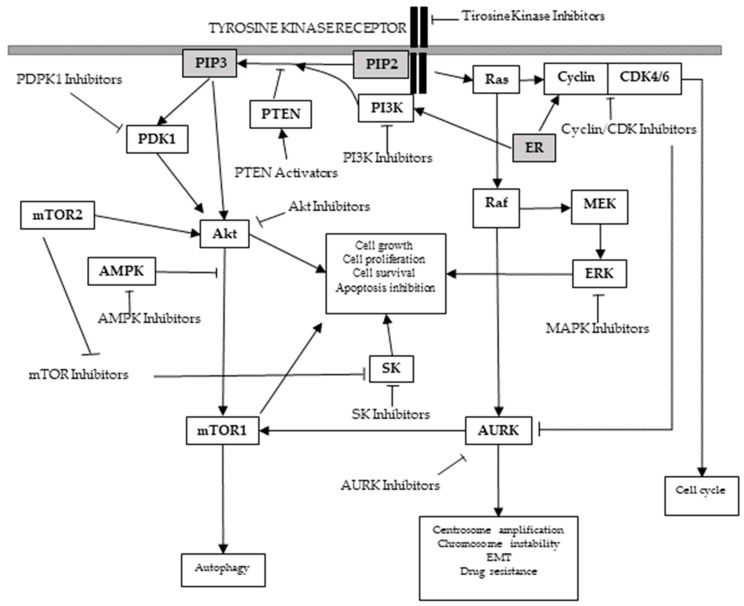
Main altered protein kinase pathways in breast cancer. (→: Activation; T: Inhibition).

Main altered protein kinase pathways and kinase targets for breast cancer treatment are presented below (Figure 3).

#### 2.3.1. PI3K/Akt/mTOR Targeting Studies

Preclinical and clinical evidence shows that a combination of PI3K/AKT/mTOR pathway inhibitors plus endocrine or trastuzumab therapy improves the clinical outcome in the treatment of ER-positive or HER2-enriched breast cancer patients, respectively [52,53]. Indeed, the proven efficacy of different protein kinases targeted activation or inhibition during ER-positive breast cancer sensitization to endocrine therapies [37,38,46,54,57,120,121,122] has led to the development of several substances, including the mTOR allosteric inhibitors rapamycin/sirolimus and the rapamycin synthetic analogs, or rapalogs, everolimus, temsirolimus, and deforolimus (Table 10).

Even though phase III trial results performed with temsirolimus in combination with letrozole [129] were not satisfactory, two clinical studies, the randomized phase II TAMRAD, and the randomized phase III BOLERO-2 trials have confirmed that a combination of the mTOR inhibitor everolimus plus tamoxifen or the aromatase inhibitor exemestane, respectively, significantly improves the clinical benefit rate, the progression-free survival, and the overall survival of ER-positive, HER2-negative postmenopausal patients, with advanced breast cancer refractory to letrozole or anastrozole [130,131]. Everolimus and trastuzumab combined therapy in metastatic HER2-enriched breast cancer patients who progressed on trastuzumab-based therapy has also been investigated in clinical trials, with positive results [132]. In spite of these encouraging results, it is not still known if the addition of everolimus to existing hormone therapy would be enough to reverse the acquired resistance [133], and whether the toxic effects of such combinations might limit the practical use of these therapeutic strategies [134].

Although the development of Akt-specific and isoform-selective inhibitors based on this kinase catalytic domain has been predicted to be difficult due to high sequence homologies [55] and mechanism-based toxicities derived from targeting the inactive Akt conformation [135], several Akt inhibitors [55,136] and three generations of PI3K inhibitors are currently in the development phase [136] with promising results. (Table 11 and Table 12).

In contrast with the positive results that were obtained with mTOR inhibitors mentioned above, and despite the important roles that Akt and PI3K activation play in breast cancer progression, no Akt or PI3K inhibitors have been approved for oncologic use yet [55]. This is in part due to the fact that the antitumor activity of PI3K inhibitors as single agents are quite limited as a result of feedback regulation and crosstalk with other signaling pathways [153].

#### 2.3.2. PTEN Pathway Targeting Studies

PTEN study’s findings offer a new approach for the development of a targeted therapy of PTEN-deficient breast carcinomas [67] and several substances, such as quercetin, that can increase PTEN levels [154], are currently under study [55]. Interestingly, PTEN restoration also sensitizes breast cancer cells to PI3K-inhibitors [155], as PTEN is in close relation with the PI3K/Akt/mTOR protein kinases acting as a PI3K antagonist by de-phosphorylating the PIP_2/3_ membrane docking sites for Akt and blocking the membrane recruitment that leads to Akt activation [58] (Figure 2).

Studies show that ER-α can induce PTEN down-regulation through PI3K activation [156] (Figure 2). As PTEN down-regulation correlates with PI3K pathway activation, leading to uncontrolled cell proliferation [67,157] and endocrine resistance [46], PI3K [158] or Akt [55] inhibition in combination with PTEN activation [157] and endocrine therapies would be useful approaches for ER-positive carcinomas with PTEN deficiency treatment [46].

It has also been shown that although adjuvant trastuzumab is beneficial for HER2-positive breast cancer patients, independently of tumor PTEN status [74,159], restoration of endogenous PTEN expression leads to marked HER2-enriched disease regression [60]. However, PTEN loss, which seems to be caused by autophagy defects [160], has been shown to correlate with a significant response to trastuzumab in early-stage HER2-positive breast cancer patients [161], while patients with advanced breast cancer will show a poor response [55,162,163], which justifies additional research in this regard.

Studies in both in vitro and in vivo models, also show that the combined use of HER2 and PI3K inhibitors in PTEN-deficient HER2-positive breast tumors is effective to reduce PI3K/Akt signaling and growth of cancer cells [164]. The continued use of trastuzumab in this type of carcinomas also induces the epithelial-to-mesenchymal transition (EMT), transforming HER2-positive to triple negative cells, and increasing the frequency of cancer stem cells and metastasis risk [165]. These transformed cells show an increased sensitivity to isothiocyanate sulforaphane compared to parental cells, which justifies the application of different treatment in these cases [165].

Finally, in vitro studies show that PI3K small-molecule inhibitors reduce the growth of dysregulated PTEN TNBC tumors [166,167], especially when combined with other drugs that are commonly used to treat advanced TNBC, like docetaxel [166].

#### 2.3.3. PDK1 Pathway Targeting Studies

Recent preclinical studies focused on PDK1 as target for breast cancer treatment show that PDK1 inhibition leads to increased anoikis and apoptosis [168], correlating more closely to antitumor activity, with minimal toxicity in xenograft models, than Akt inhibition [79]. PDK1 silencing also appears to show a synergic antitumor activity with paclitaxel [79], and to increase the sensitivity to multiple ER-α antagonists, including tamoxifen [169]. A laboratory study has also reported that the use of PDK-inhibitors with trastuzumab can reverse the trastuzumab-resistant phenotype in HER2-positive breast cancer cells [170], which justifies further studies in this regard.

##### MAPK Pathway Targeting Studies

Targeted assays against different MAPK/ERK pathway components have been recently done with variable results when used in different solid tumors [84].

As MAPK over-expression is usually found on TNBC cells, leading to tumor development and progression, and stimulating cancer stem-like cell expansion, the targeted inhibition of this protein kinase has been proposed as a novel therapeutic option for the treatment of triple negative and HR-negative breast cancer [87,88,89]. In this regard, it must be highlighted that as progesterone, androgens, and estradiol stimulate cell proliferation by means of mechanisms involving MAPK activation, strategies used for hormone dependent breast cancer treatment may lead to an increased MAPK activation and cell proliferation [86]. Recent studies have also showed that tunicamycin synergistically enhances the antitumoral effects of paclitaxel, increasing breast cancer cell apoptosis via paclitaxel-induced elevation of AKT and MAPK pathways inhibition [171].

#### 2.3.4. Cell Cycle Proteins Pathway Targeting Studies

Both cyclins and CDKs have been targeted to treat different malignancies, including breast cancer, by means of small molecules, peptides, immunotherapy, and other inhibitors [108]. In this regard, supplementation with pharmacological CDK inhibitors alone or in combination with selective antiestrogens have shown prevention of ER-α activation and control over deregulated cell cycle [110]. The role of the novel, oral, reversible CDK4/6 inhibitor, palbociclib as a potential target in ER-positive breast cancers has recently been validated in a preclinical and phase I/II clinical trial [92] (Table 13).

Both Aurora kinase A and B have been suggested as treatment targets in aromatase inhibitors-resistant cells, as therapies targeting both ER and Aurora kinases appear to be a potent strategy for overcoming aromatase inhibitors resistance in breast cancer [172]. Additional studies in xenografts also show that AURKA over-expression enhances mTOR activity under metabolic stress, suppressing autophagy [99] and increasing ER-positive breast cancer progression through EMT activation and CD44^+^/CD24^low/−^ cell-surface antigens genesis [37]. As a result of such studies, the effect of different substances, like the potent AURKA inhibitor alisertib, has been studied on PI3K/AKT/mTOR pathway, demonstrating their ability to induce in vitro inhibition of this metabolic route and promote cellular apoptosis and autophagy [173]. Among these findings, mTOR inhibition by rapamycin has been shown to sensitize AURKA-overexpressing breast cancer cells to metabolic stress-induced cell death [99]. AURKA knockdown studies also show that this protein kinase is essential for the growth of tamoxifen-resistant cells, and that its inhibition re-sensitizes tamoxifen-resistant cells to tamoxifen treatment [98].

Along with a reduction of cyclin—A expression, alisertib [174] also reduces centrosome amplification promoted by AURKA, since Cyclin-A/Cdk2 kinase activity mediates AURKA-induced centrosome amplification [174].

In vitro studies carried out with TNBC cell lines showed that alisertib is a potent pro-autophagic [173], anti-proliferative, and pro-apoptotic agent [175]. Further phase II trials of alisertib demonstrated to be effective for the treatment of breast cancer patients [176] via modulation of p38 MAPK/Akt/mTOR pathway [173]. Other different substances, such as resveratrol [177], midostaurin [178], AKI603 [179], ENMD-2076 [180] or the pan-AURKA inhibitor danusertib hydrochloride (PHA-739358) [173] are currently under in vitro and clinical study to assess their safety and activity [176,181] in TNBC or resistant breast cancer tumors treatment (Table 13).

Nowadays, a number of PLK1-inhibitors are under investigation for their use as targeted therapies against cancer [102]. The potential use of NEK2 inhibition as a targeted treatment against different types of malignancies is also under study, with promising expectations [106,182,183,184,185], as in vitro NEK2 inhibition induce breast cancer cells aneuploidy and cell cycle arrest, especially in TNBC [107].

#### 2.3.5. Sphingosine Kinase Pathway Targeting Studies

Exogenous administration of S1P apparently increases the cytotoxic potential of chemotherapy drugs docetaxel, doxorubicin, and cyclophosphamide against breast cancer metastatic cell lines [189]. The use of SK1 antagonists, which inhibits cell growth and induces apoptosis in different human cancer cell lines, also have radio-sensitizing effects on TNBC cell lines, increasing the anti-proliferative and pro-apoptotic effects that are induced by ionizing radiation [190]. SK inhibition also induces apoptosis and reduces cell proliferation in both in vivo and in vitro TNBC models [118].

mTOR inhibitor everolimus significantly inhibits SK1 and vascular endothelial growth factor (VEGF) expression, suppressing tumor growth, VEGF expression and tumor vasculature in xenograft models [191] (Table 14).

## 3. Discussion

Numerous studies have highlighted the role of aberrant protein kinases during breast carcinogenesis, cancer progression and resistance acquisition (Table 15).

Although basic research studies targeting these proteins have had satisfactory results in the hope of resulting in a new treatment in the fight against cancer, the development of new therapies based on the activation or inhibition of oncogenic protein kinases is facing numerous difficulties.

On one hand, developing new drugs that selectively act on a specific enzyme is a tough challenge, as studies show that due to the conserved kinase structure, many small molecules exhibit a high degree of promiscuity and bind to multiple kinases [192], which makes it necessary for any new substance to present a high selectivity profile. In this regard, protein crystal structures, computational molecular modeling, and docking studies have become of vital importance during the process of new kinase drug design [19] to avoid undesired effects during therapeutic use.

On the other hand, there is a rich downstream and upstream network from aberrant protein kinases that include other kinases, which are understudied and untargeted by chemical probes [12]. Crosstalk between proteins involving different kinase pathways would promote drug resistance development because of alternate kinase activation [193], which have led to the intensive study of multi-kinase inhibitors [194]. In addition to this, recent studies have shown a close relationship between microRNAs (miRNAs) activity and different protein kinase pathways, which can have an oncogenic role [69,195,196,197]. This relationship is also focus of research.

Given the above, and despite the toxic effects that may occur, combination therapy is considered the best therapeutic strategy to enhance treatment efficacy, as the use of single kinase inhibitors have only demonstrated modest clinical benefits [155,198] (Table 16).

Last, but not least, the existing heterogeneity at the kinome level across histological and within breast cancer subtypes [89] are a great impediment to apply these therapies in a generalized way to all breast cancer patients, requiring a thorough preliminary study and selection of patients that would benefit from these treatments. In this respect, precision medicine appears as a promising medical tool, since, in contrast to strategies developed for average patients, this emerging approach uses individual variability in genes, environment or patients’ lifestyle as a basis for a customized healthcare. Genomic tumor screenings, big data analytics [207], and patients’ virtual or digital models with probabilistic outputs [208], among other models, may help to predict which treatment and prevention strategies will work in each breast cancer patient and provide a valuable clinical decision support.

## Figures and Tables

**Table 1:** Proposed structures for the main protein kinase family groups.

Kinase Group	Crystal Structure	PDB ID
AGC	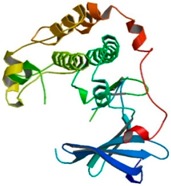	1MRV Crystal structure of an inactive Akt2 kinase domain
CAMK	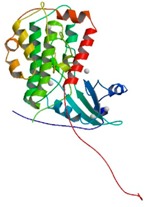	1KWP Crystal structure of MAPKAPK2
CK1	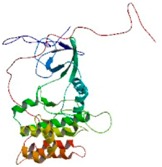	2RSV Solution structure of human full-length vaccinia related kinase 1 (VRK1)
CMGC	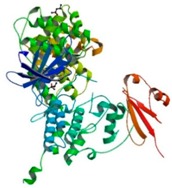	4YC3 CDK1/CyclinB1/CKS2 Apo
STE	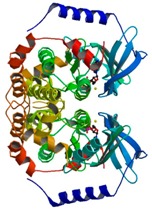	3W8Q Structure of the Human Mitogen-Activated Protein Kinase Kinase 1 (MEK1)
TK	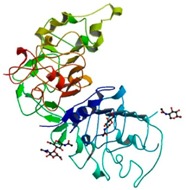	2A91 Crystal structure of ErbB2 domains 1–3
TKL	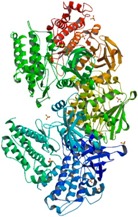	1IAS Cytoplasmic domain of unphosphorylated type I TGF-β receptor crystalized without FKBP12

Images adapted from: The Protein Data Bank [21]. www.rcsb.org.

**Table 2:** Human epidermal growth factor receptor 2 (HER2) inhibitors.

Drug	Approval Status	Structure	Adapted from
Trastuzumab (Herceptin)	Approved by the Food and Drug Administration (FDA) for the treatment of HER2-positive advanced breast cancer in combination with letrozole [29] and as part of a treatment regimen containing doxorubicin, cyclophosphamide and paclitaxel for the adjuvant treatment of women with node-positive, HER2-overexpressing breast cancer [30].	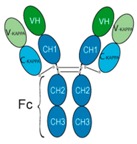	www.drugbank.ca/drugs [31]
Lapatinib (Tykeb)	Approved in 2010 by the FDA, for the treatment of HER2-positive advanced breast cancer in combination with Letrozole [29].	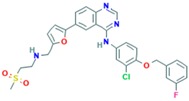	PubChem CID: 208908
Neratinib (NERLYNX)	Approved in 2017 by the FDA for the extended adjuvant treatment of adult patients with early stage of HER2-overexpressed breast cancer, to follow adjuvant trastuzumab-based therapy [32].	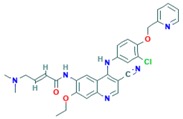	PubChem CID: 9915743
Gefitinib (Iressa)	Approved by the FDA as first-line treatment of patients with a type of metastatic lung cancer [33] and for non-small cell lung cancer patients who are currently benefiting, or have previously benefited, from gefitinib treatment [34].	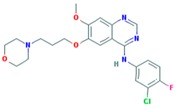	PubChem CID: 123631
Afatinib (Giotrif)	Approved by the European Commision for patients with EGFR mutation positive lung cancer [35].	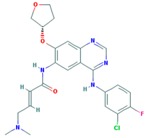	PubChem CID: 10184653

**Table 3:** Endocrine therapies for Hormone Receptor-Positive Breast Cancer treatment.

Drug	Target	Approval Status
Tamoxifen (TMX, Novaldex)	Selective Estrogen Receptor Modulator	First line treatment in pre- and post-menopausal women with ER-positive breast cancer [42]
Non steroidal: Anastrozole, letrozole	Aromatase Inhibitor	Standard therapy for post-menopausal women with ER+ breast cancer [43,44]
Steroidal: Exemestane		

**Table 4:** Altered phosphatidylinositol-3-kinase (PI3K)/Akt/ rapamycin (mTOR) pathway kinases and significance in breast cancer.

Kinase/Group	Function	Significance in Breast Cancer
KINASE: PI3K GROUP: Atypical	Converts phosphatidylinositol bisphosphate, PI(4,5)P2, to phosphatidylinositol triphosphate PI(3,4,5,)P3, which acts as a docking phospholipid site for the membrane localization of other kinases including Akt [58]. Transmits growth factor signals from receptor tyrosine kinases to down-stream mediators.	Mutations in the lipid kinase family PI3K are frequently found in breast cancer [55], occurring in up to 25% of breast cancers [59], in over 70% ER-positive breast cancer (up to 45% of luminal A and 29% of luminal B breast cancers) [38] and in 25% of HER2-positive tumors [59].
KINASE: Akt (Protein kinase B or PKB) GROUP: AGC	This serine/threonine protein kinases are one of the down-stream mediators of PI3Ks that, in turn, activates a series of other down-stream effectors that promote cellular proliferation and survival [60].	Akt over-expression is frequently observed in breast cancer [55], and has been related to lower survival rates [61]. Specifically, Akt2 isoform over-expression correlates with ER-negative and HER2-positive breast carcinomas [62], acting as a survival and anti-apoptotic factor [62] leading to enhanced tumorigenesis and metastasis. Along with the increased activity of the Akt1 isoform, which has been found in up to 40% of breast carcinomas [62], dysregulated Akt3 isoform has also been related with increased aggressiveness and poor prognosis of steroid hormone-insensitive breast carcinomas [55,62].
KINASE: mTOR GROUP: Atypical PI3K-related protein kinase family (PIKK).	This serine/threonine protein kinase is found in two structurally and functionally distinct complexes (mTORC1 and mTORC2 holoenzymes). Responsible of Akt phosphorylation and activation. Directly or indirectly regulates the phosphorylation of at least 800 proteins.	mTOR activation or increased activity has been frequently found in breast cancer and has been related to resistance to trastuzumab, endocrine therapy and cytotoxic chemotherapy [63].

**Table 5:** Phosphatase and Tensin Homologue (PTEN) alterations and significance in breast cancer.

Phosphatase	Function	Significance in Breast Cancer
PTEN	Tumor suppressor which inactivates PI3K-dependent signaling [64].	Mutated or lost in up to 44% [38,66] breast cancer patients [67]. PTEN deficiency correlates with poor prognosis [60,68], chemoresistance [69,70] and increased cell growth [67]. Low PTEN expression in TNBC is associated with early-onset breast cancer and late stage [71].

**Table 6:** PDK1 alterations and significance in breast cancer.

Kinase/Group	Function	Significance in Breast Cancer
KINASE: PDK1 GROUP: AGC kinases	Master kinase key for the activation of Akt and other AGC kinases.	PDK1 alteration is a critical component of oncogenic PI3K signaling in breast cancer [80].

**Table 7:** Mitogen-Activated Protein Kinase Pathway (MAPK) alterations and significance in breast cancer.

Kinase/Group	Function	Significance in Breast Cancer
KINASE: MAPK GROUP: CMGC	This serine/threonine kinase controls the transduction of extracellular signals to pathways related to cell growth, proliferation, differentiation, development, transformation, migration or death [81,82,83].	Involved in malignant breast cancer behavior [85]. Breast carcinomas frequently contain an increased proportion of cells with the activated form of MAPK [86]. Development of estrogen-independent growth of initially ER-α positive and hormone sensitive breast cancer cells [37]. MAPK over-expression is usually found on TNBC cells, leading to tumor development and progression and stimulating cancer stem-like cell expansion [87,88,89].

**Table 8:** Cell cycle proteins alterations and significance in breast cancer.

Kinase/Group	Function	Significance in Breast Cancer
KINASE: CDK GROUP: CMGC kinases	Cell cycle regulation [92].	Suggested to play a role in hormone receptor positive breast cancer [93].
KINASE: Aurora Kinases GROUP: AUR branch, near AGC group	Control the accurate and equal segregation of genomic material during mitosis [91].	Over-expressed in breast cancer and other malignancies [91]. AURKA (Aurora Kinase A) over-expression promotes tumor formation [94,95], EMT (epithelial to mesenchymal transition) activation [96], metastasis [94,95,97], drug resistance [37,97,98], endocrine resistance [37,96], autophagic cell death resistance, enhanced breast cancer cell survival when exposed to metabolic stress [99] and a worse prognosis in ER-positive [95,96,100] and TNBC breast carcinomas [101]. AURKA signaling is highly correlated to TNBC [37,101]. Aurora kinase B is associated with reduced disease-free and overall survival of patients who have received tamoxifen as first-line adjuvant endocrine treatment, and has been suggested as a driving factor of antiestrogen resistant breast cancer cell models’ growth and as a biomarker for reduced benefit of tamoxifen treatment [100].
KINASE: PLK1 GROUP: Other kinases	Initiation, maintenance, completion of mitosis [102]. Maintenance of genomic stability [103].	PLK1 signaling cooperates with estrogen receptor-dependent transcription [104]. Related to hormone-independent, ER-positive breast cancer [105].
KINASE: NEK2 GROUP: Other kinases	Involved in the regulation of centrosome duplication and spindle assembly during mitosis [106].	Related to cell growth and aneuploidy in breast cancer cells [107].

**Table 9:** Sphingosine kinases alterations and significance in breast cancer.

Kinase/Group	Function	Significance in Breast Cancer
KINASE: SK GROUP: AGC	These serine/threonine kinases catalyze sphingosine into sphingosine-1-phosphatase phosphorylation.	Associated with breast cancer progression and resistance to drug therapies [116,117,118]. Predictive of poor prognosis in human breast cancer [119].

**Table 10:** mTOR Inhibitors.

Drug	Approval Status	Structure	Adapted from
Rapamycin/Sirolimus	Approved by the FDA to treat lymphangioleiomyomatosis [123].	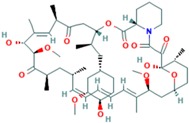	PubChem CID: 5284616
Everolimus (Afinitor)	Approved by the FDA for the treatment of adult patients with progressive, well-differentiated non-functional, neuroendocrine tumors of gastrointestinal or lung origin with unresectable, locally advanced or metastatic disease [124], advanced HR-positive, HER2-negative breast cancer, advanced renal cell carcinomas, subependymal giant cell astrocytoma and renal angiomyolipomas associated with tuberous sclerosis [125].	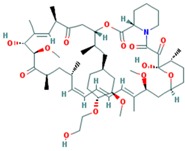	PubChem CID: 6442177
Temsirolimus	Approved by the FDA for the treatment of advanced renal cell carcinoma and under study for other types of malignancies including breast cancer [126].	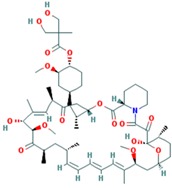	PubChem CID: 6918289
Deforolimus	Investigational oral mTOR inhibitor in development for the treatment of metastatic soft-tissue or bone sarcomas [127].	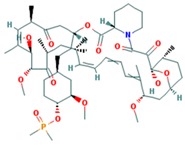	PubChem CID: 102284657
Sapanisertib (1224844-38-5, HY-13328, INK-128, MLN-0128, TAK-228)	mTOR small molecule inhibitor in phase II clinical trials for breast cancer, endometrial cancer, glioblastoma, neuroendocrine tumors, renal cell carcinoma, soft sarcoma and thyroid cancer [128]	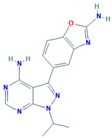	PubChem CID: 45375953

**Table 11:** PI3K inhibitors.

Drug	Approval Status	Evidences	Structure	Adapted from
GSK2636771	Under study	Potential antineoplastic activity resulting in tumor cell apoptosis and growth inhibition in PI3K β-expressing and/or PTEN-driven tumor cells [137].	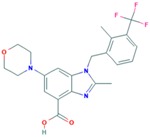	PubChem CID: 56949517
Buparlisib (NVP-BKM120, 944396)	Under study	Partially overcomes multidrug resistance phenotype in chemoresistant breast cancer cells [138]. Significantly inhibits TNBC cell lines proliferation [139]. Phase II trial of single agent BKM120 in patients with TNBC metastatic breast cancer [140].	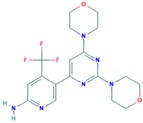	PubChem CID: 16654980

**Table 12:** Akt inhibitors.

Drug	Approval Status	Evidences	Structure	Adapted from
Miltefonsine (Impavido)	FDA approved tropical disease leishmaniasis treatment	Has proven to be effective and tolerable as a local treatment for cutaneous metastasis from breast cancer [141,142,143].	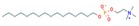	PubChem CID: 3599
Perifosine (KRX-0401)	Under study	Although no objective responses were seen in the phase II trial tested on a group of pretreated metastatic breast cancer patients [144], it has recently been suggested that Perifosine can restore the sensitivity to tamoxifen [145] and reverse the P-glycoprotein-mediated multidrug resistance in vitro [146], so further research is needed.	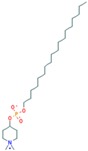	PubChem CID: 148177
AZD5363	AZD5363 is undergoing clinical assays phase I and II [55].	This pan-Akt catalytic inhibitor has been shown to decrease the proliferation of resistant breast ER-positive cancer cell lines, to re-sensitize model breast cancer cells to tamoxifen [147] and to enhance the antitumor activity of docetaxel, lapatinib and Trastuzumab in breast cancer xenografts [55].	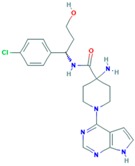	PubChem CID: 25227436
Ipatasertib (GDC-0068)	Under study	This novel selective ATP-competitive small molecule inhibitor has proven to preferentially target the active phosphorylated Akt isoform and to have antitumor activity in solid tumors with activation of Akt [135]. The combination of ipatasertib plus paclitaxel has been studied in a phase II trial as first-line therapy for metastatic TNBC with positive results [148].	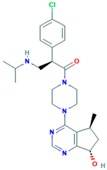	PubChem CID: 124652937
MK-2206	Under study	In accordance with preclinical data, the phase I clinical trial concluded that the combination of this allosteric pan-Akt inhibitor with the HER2-targeted drug lapatinib may be a promising approach to overcome resistance to treatment [149]. Despite the positive results of phase I trial testing MK-2206 in combination with anastrozole [150], phase II trial has concluded that the combined treatment of MK-2206 with hormonal therapy (anastrozole) in PIK3CA-mutant, ER-positive and HER2-negative breast cancer patients does not provide clinical benefit [151]. A recent study has shown that breast cancer cells can acquire resistance to MK2206 through the over-expression of Akt3 [152]. Apparently, this chemoresistance can be reversed by the inhibition of Akt3 [152] which should be taken into consideration during MK2206 phase II neoadjuvant trials.	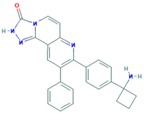	PubChem CID: 24964624

**Table 13:** Cell Cycle Proteins Inhibitors.

Drug	Target	Approval Status	Structure	Adapted from
Palbociclib	CDK4/6	FDA approved for the treatment of hormone receptor positive, HER2 negative advanced or metastatic breast cancer in combination with an aromatase inhibitor as initial endocrine based therapy in postmenopausal women [186].	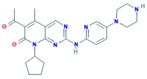	PubChem CID: 5330286
Alisertib	AURK	Being evaluated in multiple clinical trials in both solid cancers (neuroblastoma, small cell lung cancer, neuroendocrine prostate cancer, atypical teratoid/rhabdoid tumors, and breast cancer among others) and heme-lymphatic malignancies [187].	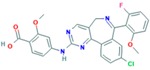	PubChem CID: 24771867
Resveratrol	AURKA, PLK	Under study [177].	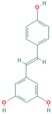	PubChem CID: 445154
Midostaurin (PCK412)	AURKA	Currently approved by the FDA for the treatment of adult patients with newly diagnosed acute myeloid leukemia, who are FLT3 mutation-positive, in combination with standard cytarabine and daunorubicin induction and cytarabine consolidation [188].	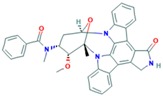	PubChem CID: 9829523
ENMD-2076	AURK	Under study [180].	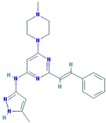	PubChem CID: 16041424
Danusertib	AURK	Under study [173].	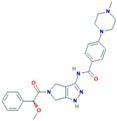	PubChem CID: 11442891

**Table 14:** Sphingosine kinase inhibitors.

Drug	Target	Approval Status	Structure	Adapted from
Sphingosine kinase inhibitor (SK inhibitor, 1177741-83-1)	SK	Investigational	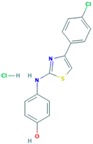	PubChem CID: 16760659

**Table 15:** Altered expression of kinases in different breast cancer subtypes.

Breast Cancer Subtype	Altered Kinase	References
HER2-Enriched	HER2	[26]
	PI3K/Akt/mTOR	[53]
	PI3K	[59]
	mTOR	[63]
	PTEN	[60]
	PDK	[170]
Estrogen Receptor-Negative HER2-Enriched	Akt	[62]
Estrogen Receptor-Positive HER2-Negative	mTOR	[125]
Hormone Receptor-Positive	PI3K/Akt/mTOR	[52]
	PI3K	[38]
	Akt	[55,62]
	mTOR	[63]
	PDPK1	[169]
	MAPK	[37]
	AURK	[37,95,96,100]
	CDK	[93]
	PLK1	[104]
Basal-Like	PTEN	[71]
	MAPK	[87,88,89]
	AURK	[37,101]
	SK	[118]

**Table 16:** Combination therapy strategy with protein kinases for breast cancer treatment.

Targeted Kinase	Combined with	Breast Cancer Patients That Would Benefit	Trials
PI3K	Hormonal therapy	ER-positive, metastatic	Phase I trial of buparlisib in combination with fulvestrant [199]
mTOR	Hormonal therapy	ER-positive, HER2-negative, metastatic	Phase II trial of everolimus in combination with tamoxifen [131]
	Aromatase Inhibitor	ER-positive, metastatic	Phase III trial of everolimus in combination with exemestane [130]
	Trastuzumab	Trastuzumab-resistant and taxane-pretreated, HER2-positive, metastatic	Phase III trial of everolimus in combination with trastuzumab plus vinorelbine [132]
Akt	Chemotherapy	TNBC	Phase II trial of ipatasertib plus paclitaxel [148]
PTEN	PI3K Inhibitor	TNBC with PTEN deficiency	Phase I/IIa study of GSK2636771 [200]
PDK1	Trastuzumab	HER2-enriched	Preclinical study in combination with [170]
	Chemotherapy	Breast cancer patients	Preclinical study in combination with with paclitaxel [79]
	Hormonal therapy	ER-positive	Preclinical study in combination with tamoxifen [169]
	CDK4/6 inhibitors	Resistant to CDK4/6 inhibitors	Preclinical study in combination with ribociclib [201]
MAPK	Hormonal therapy	ER-positive, metastatic	Phase I study of p38 MAPK inhibitor, ralimetinib, in combination with tamoxifen [202]
	Chemotherapy	Advanced TNBC	Phase II study of MEK inhibitor, cobimetinib, in combination with paclitaxel [203]
CDK	Hormonal therapy	ER-positive, HER2-negative, metastatic	Phase III trial of fulvestrant in combination with palbociclib [204]
	Anti-HER2 plus endocrine therapy	ER-positive, HER2-positive, metastatic	Ongoing clinical trial with palbociclib [205]
AURK	Hormonal therapy	ER-positive, metastatic or locally advanced	Ongoing clinical trial of alisertib and fulvestrant [206]
	Small kinase inhibitor	Metastatic TNBC	Ongoing clinical trial of alisertib and sapanisertib [137]
